# Tofacitinib treatment of systemic juvenile idiopathic arthritis: a case report and literature review

**DOI:** 10.3389/fped.2025.1482762

**Published:** 2025-02-13

**Authors:** Meifang Zhu, Yan Zhao, Xiaohua Zhang, Peng Zhou, Jing Jin, Zhidan Fan, Haiguo Yu

**Affiliations:** ^1^ Department of Rheumatology and Immunology, Children’s Hospital of Nanjing Medical University, Nanjing, China; ^2^ Department of Ultrasonography, Children’s Hospital of Nanjing Medical University, Nanjing, China

**Keywords:** tofacitinib, systemic juvenile idiopathic arthritis, effectiveness, treatment, JAK

## Abstract

**Objective:**

Systemic juvenile idiopathic arthritis (sJIA), a particularly aggressive form of childhood arthritis, is characterized by persistent systemic inflammation. The most advanced treatments include biologic agents that target the interleukin-1(IL-1) and interleukin-6(IL-6) pathways. However, sJIA continue to pose challenging challenges for rheumatologists treating pediatric patients worldwide.

**Methods:**

1 children with sJIA was retrospectively collected from the Department of Rheumatology and immunology, Children's Hospital of Nanjing Medical University, Nanjing. Literature published between 2019 and 2024 was reviewed to understand the effect of tofacitinib on patients with sJIA.

**Results:**

After a month of treatment of tofacitinib, there was a significant improvement in clinical symptoms and inflammatory indicators showed a marked decrease. As of July 2023, the patient's condition was effectively in remission. The efficacy of tofacitinib treatment was remarkable.

**Conclusion:**

Tofacitinib has shown good efficacy and safety in the treatment of sJIA patients, effectively controlling disease activity and relieving symptoms. The application of Janus kinase (JAK) inhibitors may offer a new treatment option for this disease.

## Introduction

Juvenile idiopathic arthritis (JIA) is the most prevalent rheumatic condition of unknown etiology in pediatric populations, primarily characterized by peripheral arthritis (1). It represents a major cause of acquired disability among children (2). Systemic juvenile idiopathic arthritis (sJIA), one of the predominant subtypes of JIA, is distinguished by severe systemic inflammation and is frequently associated with arthritis (3). In recent years, the therapeutic application of IL-1 and IL-6 inhibitors has resulted in remission in a considerable proportion of patients (60%–80%) (4). However, despite these advancements, the frequent requirement for subcutaneous or intravenous administration poses substantial challenges for young patients.

Tofacitinib, as the first-generation Janus kinase (JAK) inhibitor, has been employed in the treatment of rheumatoid arthritis (5). It exerts its therapeutic effects by inhibiting JAK1 and JAK3, thereby disrupting the JAK-STAT signaling pathway. This inhibition effectively blocks the transmission of inflammatory cytokines, leading to significant improvements in disease symptoms and reductions in structural joint damage. However, there is limited literature on the application of tofacitinib in systemic juvenile idiopathic arthritis (sJIA) within China. In this report, we present our experience with tofacitinib in the treatment of sJIA.

## Case presentation

In April 2018, a 9-year-old girl visited our hospital presenting with recurrent fever accompanied by multiple joint swelling and pain.

One year prior, the patient experienced a fever reaching 40°C along with arthritis affecting the left knee and right ankle. During periods of high fever, a dense erythematous rash appeared on the neck, abdomen, and bilateral thighs, accompanied by pruritus, which resolved upon defervescence. Laboratory findings revealed significantly elevated levels of C-reactive protein (CRP), white blood cells (WBC), neutrophils (NE), erythrocyte sedimentation rate (ESR), and serum ferritin (SF). Systemic juvenile idiopathic arthritis (sJIA) was suspected. From September 2017 to April 2018, the patient sought treatment at multiple hospitals, receiving various therapies including prednisone (15 mg orally twice daily), naproxen (0.15 g orally twice daily), and leflunomide (10 mg orally daily). Due to liver function impairment, oral prednisone was subsequently replaced with methylprednisolone (8 mg orally twice daily). Despite these interventions, there was minimal improvement in her fever and arthritis symptoms.

With recurrent fever and arthritis, the patient was admitted to our rheumatology department for initial evaluation in April 2018. On physical examination, her left knee and right ankle exhibited warmth, swelling, and tenderness upon palpation. She was non-compliant during Patrick's test. Laboratory investigations revealed elevated white blood cell (WBC) count of 14.71 × 10^9^/L (normal: 4.1–11.0 × 10^9^/L), neutrophil (NE) count of 10.33 × 10^9^/L (normal: 2.0–7.0 × 10^9^/L), erythrocyte sedimentation rate (ESR) of 50 mm/h (normal: <15 mm/h), C-reactive protein (CRP) level of 78.00 mg/L (normal: <8.0 mg/L), serum ferritin (SF) level of 991.4 ng/ml (normal: 11–306.8 ng/ml), interleukin-6 (IL-6) level of 52.4 pg/ml (normal: <5.4 pg/ml), interleukin-1β (IL-1β) level of 8.27 pg/ml (normal: <5.0 pg/ml), and tumor necrosis factor α (TNF-α) level of 21.9 pg/ml (normal: <8.5 pg/ml). Serum creatinine, transaminase, lipid profile, and procalcitonin levels were within normal limits. Rheumatoid factor, antinuclear antibody, anti-cyclic citrullinated peptide antibodies, and HLA-B27 tests were negative. Ultrasound examination of the neck revealed multiple hypoechoic lymph nodes, suggestive of inflammatory changes. Echocardiography did not reveal any significant abnormalities. Based on the clinical presentation, laboratory findings, and imaging results, the patient met the diagnostic criteria for systemic juvenile idiopathic arthritis (SJIA) as defined by ILAR in 2001. Treatment was initiated with methylprednisolone (1 mg/kg IV), leflunomide, methotrexate (8 mg/m^2^ PO weekly), thalidomide (25 mg PO BID), and tocilizumab (TCZ, 6 mg/kg IV) to manage her arthritis and systemic inflammation. After two years of treatment, both her systemic symptoms and arthritis significantly improved.

Subsequently, the patient received tocilizumab (TCZ) on a bimonthly basis, leading to continued improvement in her condition without fever recurrence and gradual alleviation of arthritic symptoms. However, in July 2021, the patient exhibited limited extension and movement of the left elbow joint along with tenderness. Laboratory tests revealed elevated levels of CRP (74.00 mg/L) and ESR (76 mm/h). Given the recurrent nature of the condition and the lack of sustained relief following multiple treatments, it was decided, after thorough discussion with the parents, to introduce the JAK inhibitor tofacitinib (5 mg po bid) in July 2021. The final treatment regimen was adjusted to include tofacitinib in combination with TCZ and methotrexate.

**Figure 1:**
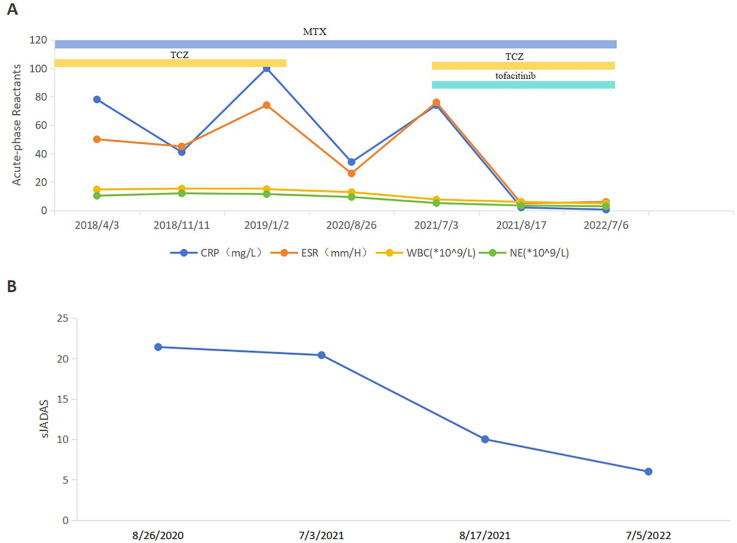
Follow-up of disease activity and acute-phase reactant levels of the patient. **(A)** ESR, CRP, WBC and NE levels before and after treatment of tofacitinib. ESR, erythrocyte sedimentation rate; CRP, C-reactive protein; WBC, white blood cell; NE, neutrophil. **(B)** sJADAS27 before and after treatment of tofacitinib. sJADAS27, systemic juvenile arthritis disease activity score 27.

Compared to the combination treatment prior to the introduction of tofacitinib, all inflammatory indices demonstrated a significant reduction, particularly C-reactive protein (CRP) levels, which decreased from 74.00 mg/L to 2.07 mg/L, and erythrocyte sedimentation rate (ESR), which dropped from 76 mm/h to 5 mm/h. Post-treatment, there was marked improvement in joint swelling, pain, and range of motion. The systemic Juvenile Arthritis Disease Activity Score (sJADAS27) also decreased from 20.4 to 10 points (Figures 1A,B). As of July 2023, the patient's condition has shown sustained and stable improvement, with the disease largely under control.

## Discussion and conclusion

In this report, we describe the case of a 9-year-old female patient with systemic juvenile idiopathic arthritis (sJIA) who was treated with tofacitinib. Initially, she received a combination therapy including prednisone, methotrexate, thalidomide, naproxen, and tocilizumab (TCZ), which effectively alleviated her acute systemic symptoms. Following the initiation of tofacitinib treatment, there was a marked improvement in her arthritis symptoms, and the levels of inflammatory markers and various cytokines progressively normalized.

Despite recent advances in treatment options, sJIA remains one of the most challenging pediatric rheumatologic conditions. Unlike other forms of childhood arthritis, sJIA is characterized by prominent quotidian fever. The disease exhibits significant heterogeneity; some children find it particularly difficult to achieve clinical remission through pharmacological interventions, and a small proportion experience joint progression that can lead to deformity and high morbidity (6). Consequently, earlier and more aggressive immunotherapy is often required to control the disease. Historically, the first-line treatment for sJIA has primarily consisted of non-steroidal anti-inflammatory drugs (NSAIDs) and systemic corticosteroids, with the latter serving as the cornerstone of initial therapy. In cases where patients are resistant to conventional treatments, timely introduction of biologics is recommended (7). The clinical approval of IL-1 antagonists and IL-6 antagonists has ushered in a new era of biological treatment for sJIA. Additionally, small molecule targeted Janus kinase (JAK) inhibitors such as tofacitinib and ruxolitinib have demonstrated efficacy in reducing systemic inflammatory responses in children with sJIA.

Tofacitinib is an oral small-molecule drug and belongs to the JAK inhibitor (8). It can selectively inhibit the activity of JAK1 and JAK3, blocking the signal transduction of various inflammatory cytokines and exerting a certain regulatory effect on T cells, which can reduce the differentiation and activation of Th1 and Th17 cells. Additionally, tofacitinib can inhibit the production of multiple inflammatory mediators, thereby alleviating joint inflammation in sJIA patients (9, 10). Although tofacitinib has been widely prescribed for adults with RA, the data on sJIA are limited.

In 2019, HUANG et al. (11) reported that a 13-year-old female patient with sJIA was treated with tofacitinib, but the disease recurred after multiple treatments. When the treatment regimen was adjusted to tofacitinib (5 mg, po, twice daily) combined with methylprednisolone (4 mg, po, qd) for six months, the patient achieved significant disease remission for the first time. The reduction and withdrawal of steroids were successfully accomplished. ZHANG et al. (12) reported a 4-year-old girl with sJIA who received multiple treatments (dexamethasone, ibuprofen, etanercept, and TCZ). Her body temperature and arthritis symptoms gradually stabilized, yet the levels of cytokines and CRP remained higher than normal values. After 5 months of treatment with tofacitinib instead of TCZ, the joint symptoms vanished, and the levels of acute phase reactants and cytokines decreased to the normal range. In this case, the patient's condition was not effectively controlled after various regimens before admission, and the condition was alleviated after the administration of tofacitinib combination treatment.

While tofacitinib has recently been approved by the FDA for the treatment of polyarticular course JIA, and there is an ongoing clinical trial for sJIA with systemic features (NCT02592434), currently only case reports support the use of these medications for sJIA (13). As can be seen from the table, in all case reports, patients treated with tofacitinib achieved good outcomes (Table 1). In addition to the efficacy of tofacitinib, its optimal therapeutic dose remains to be determined. An experiment indicates that the final median dose of tofacitinib in their series is 5 (3.75–10) mg twice a day. Moreover, higher doses of tofacitinib may contribute to a better clinical response for a significant proportion of patients in the cohort, especially those with active systemic disease (14).

**Table 1:** Treatment of sJIA patients with JAK inhibitors published between 2019 and 2024 in pubmed.

	Gender	Age	Concomitant treatments at JAKi initiation	JAKi dosing	Remaining symptoms at last follow-up	Remaining abnormal laboratory findings at last follow-up	Other adverse side effects at JAKi duration
Case 1 (11)	Female	13	Methylprednisolone (4 mg/day)	Tofacitinib 2.5 mg bid	No	ESR↑	No
Case 2 (12)	Female	4	MTX	Tofacitinib 2.5 mg bid	No	No	No
Case 3 (14)	Female	7	Prednisone (10 mg/day), MTX, TCZ	Tofacitinib 2.5 mg bid → 3.75 mg bid	Rash	CRP↑	No
Case 4 (14)	Female	13	Prednisone (6.25 mg/day), MTX, NSAID	Tofacitinib 2.5 mg bid → 10 mg bid	Occasional arthralgia	CRP↑	No
Case 5 (14)	Male	14	Prednisone (15 mg/day), MTX, TCZ	Tofacitinib 2.5 mg bid → 5 mg bid	No	No	No
Case 6 (14)	Male	12	Prednisone (12.5 mg/day), MTX	Tofacitinib 2.5 mg bid → 7.5 mg bid	Occasional fever and arthralgia	WBC↑, FER↑, CRP↑	No
Case 7 (14)	Female	16	Prednisone (5 mg/day), MTX, TCZ	Tofacitinib 2.5 mg bid → 5 mg bid	No	No	No
Case 8 (14)	Male	13	MTX, TCZ	Tofacitinib 5 mg qd → 5 mg bid	No	No	No
Case 9 (15)	Male	10	None	Tofacitinib 11 mg/day	No	CRP↑	No

JAKi, Janus kinase inhibitors; WBC, white blood cells; FER, ferritin; MTX, methotrexate; CRP, C-reactive protein; ESR, erythrocyte sedimentation rate; TCZ, tocilizumab.

In 2019, JAK inhibitors were regarded as being on a par with bDMARDs in terms of efficacy and safety. The infection rates other than herpes zoster were higher with tofacitinib compared to TNF inhibition (16). In related experiments of rheumatoid arthritis, the risk of infection of tofacitinib is similar to that of other biological agents (17). No adverse effects have occurred in this patient after treatment with tofacitinib until July 2023.

In conclusion, tofacitinib combination therapy shows better efficacy and fewer adverse reactions in treating sJIA patients. However, this experience is currently limited to a few case reports, and its exact efficacy, administration timing and sequence still need further exploration. In the future, the application of JAK inhibitors may offer a new treatment option for this disease.

## Data Availability

The original contributions presented in the study are included in the article/Supplementary Material, further inquiries can be directed to the corresponding authors.
